# Long Non-coding RNA Aerrie Controls DNA Damage Repair via YBX1 to Maintain Endothelial Cell Function

**DOI:** 10.3389/fcell.2020.619079

**Published:** 2021-01-11

**Authors:** Tan Phát Pham, Diewertje I. Bink, Laura Stanicek, Anke van Bergen, Esmee van Leeuwen, Yvonne Tran, Ljubica Matic, Ulf Hedin, Ilka Wittig, Stefanie Dimmeler, Reinier A. Boon

**Affiliations:** ^1^Department of Physiology, Amsterdam UMC, Vrije Universiteit Amsterdam, Amsterdam, Netherlands; ^2^Institute of Cardiovascular Regeneration, Goethe University, Frankfurt, Germany; ^3^Vascular Surgery Division, Department of Molecular Medicine and Surgery, Karolinska Institutet, Stockholm, Sweden; ^4^German Center for Cardiovascular Research DZHK, Partner Site Frankfurt Rhine-Main, Berlin, Germany

**Keywords:** LncRNA – long non-coding RNA, endothelial cell, aging, DNA damage, cardiovascular disease

## Abstract

Aging is accompanied by many physiological changes. These changes can progressively lead to many types of cardiovascular diseases. During this process blood vessels lose their ability to maintain vascular homeostasis, ultimately resulting in hypertension, stroke, or myocardial infarction. Increase in DNA damage is one of the hallmarks of aging and can be repaired by the DNA signaling and repair system. In our study we show that long non-coding RNA Aerrie (linc01013) contributes to the DNA signaling and repair mechanism. Silencing of Aerrie in endothelial cells impairs angiogenesis, migration, and barrier function. Aerrie associates with YBX1 and together they act as important factors in DNA damage signaling and repair. This study identifies Aerrie as a novel factor in genomic stability and as a binding partner of YBX1 in responding to DNA damage.

## Introduction

Cardiovascular disease (CVD) is the main cause of death worldwide at old age (Benjamin et al., 2019). Structural and functional changes of the vasculature accumulate throughout life resulting in increased arterial thickening, stiffness, and dysfunctional endothelium (Lakatta and Levy, 2003a). These changes result in increased systolic pressure, increased risk of development of atherosclerosis, myocardial infarction, hypertension, and stroke (Lakatta and Levy, 2003b). Aging is causally linked with many cellular changes leading to replicative senescence and inflammation. Endothelial cell senescence is associated with reduced activity of endothelial nitric oxide synthase, less responsiveness to hemodynamic forces, and inflammation (Wang et al., 2007; Kang et al., 2009; Collins and Tzima, 2011). Senescence begins with shortening of telomeres that ultimately exposes an uncapped free double-stranded chromosome end leading to activation of the DNA damage response pathway (Childs et al., 2015). Understanding the mechanisms may open new paths for intervention of CVDs.

The DNA damage response pathway is a well described and is important to maintain the genomic integrity of the aging cell. In response to DNA damage, multiple intra-cellular signaling cascades are activated in order to coordinate the appropriate repair of DNA damage (Shiloh and Ziv, 2013; Blackford and Jackson, 2017). This so-called DNA damage response (DDR) is controlled by three phosphoinositide 3-kinase (PI3K)-related kinases: Ataxia-telangiectasia mutated (ATM), ATM- and Rad3- related (ATR), and DNA-dependent protein kinase (DNA-PK) (Blackford and Jackson, 2017). Double-strand breaks (DSBs) can be recognized by the Ku70/80 heterodimer, leading to recruitment and activation of DNA-PK promoting non-homologous end joining (NHEJ) (Davis et al., 2014). DSBs are also recognized by the Mre11–Rad50–Nbs1 (MRN) complex leading to either homologous recombination which is not error-prone or NHEJ (Stracker and Petrini, 2011). Capture of DNA ends by the MRN complex leads to the rapid autophosphorylation of ATM (Bakkenist and Kastan, 2003). Once activated, ATM phosphorylates histone protein H2AX at serine 139 at the site of the DSB. H2AX is associated with the recruitment of necessary DNA repair factors to the site of DSB (Paull et al., 2000; Celeste et al., 2002). Additionally, ATM phosphorylates cell-cycle checkpoint protein CHK2 (Marechal and Zou, 2013). Both ATM and CHK2 are able to phosphorylate P53, thereby inhibiting the interaction of P53 with ubiquitin ligase MDM2, resulting in rapid P53 stabilization (Chehab et al., 2000). Due to this stabilization, P53 can control cell fate decisions, such as cell-cycle arrest, DNA repair, apoptosis, senescence, and metabolic reprogramming (Li et al., 2012; Moulder et al., 2018). Long non-coding RNAs (lncRNAs) are known to be involved in DNA repair. Unraveling their implication in DNA repair opens further possibilities in understanding the mechanisms underlying CVD (Thapar, 2018; Haemmig et al., 2020).

Long non-coding RNAs are longer than 200 nucleotides, transcribed by RNA pol-II, and are not translated into protein. These transcripts share several properties with protein coding RNAs, being 5’capped, spliced and 3’ polyadenylated (Guttman et al., 2009). In contrast, some lncRNAs such as MALAT1 and MEN β are not polyadenylated, but a highly conserved triple helical structure is generated at the 3’ end to stabilize their structure (Wilusz et al., 2012). LncRNAs contribute to a variety of physiological and pathophysiological cellular processes in the cardiovascular system (Uchida and Dimmeler, 2015). For example, they influence vessel outgrowth, remodeling, and angiogenesis by regulation of cellular processes like proliferation, apoptosis, and migration (Simion et al., 2019). The molecular functions of lncRNAs are diverse and dependent on the subcellular location. In the nucleus, lncRNAs are described as post-transcriptional regulators by binding to chromatin modifiers. Moreover, they regulate transcription by binding to transcription factors, mRNA splicing by binding to pre-mRNAs, and other various processes by binding to ribonucleic protein components. In the cytoplasm lncRNAs regulated microRNA function by acting as a miRNA sponge or they can bind to mRNA to alter its stability (Boon et al., 2016).

We identified the novel lncRNA Aerrie and provide experimental evidence for its involvement in the DNA damage repair pathway in the cardiovascular system. We show that Aerrie is expressed in the endothelium and is upregulated during aging. Loss of Aerrie reduces endothelial function due to increased DNA damage. Aerrie is associated with Y-Box protein 1 (YBX1) and both are crucial for DNA damage repair. Overexpression of Aerrie increased the efficiency of DNA repair upon genotoxic damage. Together, these results show a crucial role of lncRNA Aerrie in regulating endothelial cell aging.

## Materials and Methods

### Cell Culture

Human umbilical vein endothelial cells (HUVECs) were purchased from Lonza (batch p1028 and p1032) and cultured in endothelial cell medium (ScienceCell, 1001), supplemented with ECGS (ScienceCell, 1052), penicillin/streptomycin (ScienceCell, 0503), and 5% fetal bovine serum (ScienceCell, 0025). HUVECs were used between passage 1 and 5 for experiments unless stated otherwise. HUVECs were stimulated with unidirectional flow with 20 dyn/cm^2^ for 72 h to induce laminar shear stress and oscillatory flow with 20 dyn/cm^2^ for 14 h to induce turbulent flow. For endothelial-to-mesenchymal (endMT) experiments, HUVECs were stimulated with IL-1β and TGF-β2 for 72 h. Hek293T cells were purchased from ATCC and cultured in Dulbecco’s Modified Eagle Medium (Thermo Fisher, 31966021) supplemented with 10% FCS, 1% Pyruvate, 1% D-glucose, and 1% penicillin/streptomycin, 1% minimum essential media non-essential amino acid mix (Sigma-Aldrich, M7145). Cells were cultured at 37°C with 5% CO_2_. Cell numbers were determined with the Countess II cell counter (Thermo Fisher). All cell types were cultured at 37°C in a 5% CO_2_ atmosphere and tested negative for mycoplasma.

### BiKE

Patients undergoing surgery for symptomatic or asymptomatic, high-grade (>50% NASCET) (Naylor et al., 2003) carotid stenosis at the Department of Vascular Surgery, Karolinska University Hospital, Stockholm, Sweden, were enrolled in the Biobank of Karolinska Endarterectomies (BiKE) study. Symptoms of plaque instability were defined as transitory ischemic attack, minor stroke, and amaurosis fugax. Patients without qualifying symptoms within 6 months prior to surgery were categorized as asymptomatic and indication for carotid endarterectomy based on results from the Asymptomatic Carotid Surgery Trial (ACST) (Halliday et al., 2010). Carotid endarterectomies (carotid plaques) were collected at surgery (totally 127) and normal artery controls were obtained from nine macroscopically disease-free iliac arteries and one aorta from organ donors without any history of cardiovascular disease (totally 10). All samples were collected with informed consent from patients or organ donors’ guardians. All human studies were approved by the regional Ethics Committee. The BiKE study follows the principles outlined in the declaration of Helsinki. The BiKE study cohort demographics, details of sample collection, processing, and large-scale profiling analyses were previously extensively described (Perisic et al., 2013, 2016). The microarray dataset is available from Gene Expression Omnibus (GSE21545). Global gene expression profiles have been analyzed by Affymetrix HG-U133 plus 2.0 Genechip microarrays in 127 patients’ plaque tissues (*n* = 87 symptomatic and *n* = 40 asymptomatic) and *n* = 10 non-atherosclerotic (normal) arteries.

### ISHD Samples

Left ventricular tissues from ISHD samples were acquired from the University of Sydney (Sydney, NSW, Australia), with the ethical approval of the Human Research Ethics Committee (number 2012/2814). Explanted left ventricular heart tissue of healthy donors was used as control samples; the donors died from a non-cardiac cause, typically motor vehicle accidents. These healthy donor samples were also acquired from the University of Sydney. Patient information are shown in Supplementary Table 1.

### RT-qPCR

Total RNA from cultured HUVECs was isolated with direct-zol RNA miniprep (Zymo Research, R2052) according to the manufacturer’s protocol. For Real Time Quantitative PCR (RT-qPCR) analysis, 100–1000 ng total RNA was reverse transcribed using iScript cDNA synthesis Kit (Bio-Rad, #1708891). RT-qPCR was performed with iQ SYBR Green Supermix (Bio-RAD, #170-8886) in the Bio-Rad CFX96 Touch Real-time PCR Detection system. Ribosomal protein, large, P0 (RPLP0) or glyceraldehyde-3-phosphate dehydrogenase (GAPDH) were used for normalization of the samples. Gene expression analysis was done using the 2^–Δ^ ^CT^ method. Primer sequences are listed in the Supplementary Table 2.

### Nuclear and Cytoplasmic Cell Fractionation

Nuclear and cytoplasmic RNA fractions were separated as described in the literature (Gagnon et al., 2014). All steps were performed on 4°C. Briefly, HUVECs were collected by cell scraping and centrifugation (5 min at 500 × *g*, 4°C). Cell pellets were treated with cytoplasmic lysis buffer [10 mM Tris–HCl pH 7.5, 10 mM NaCl, 3 mM MgCl_2_, 0.5% Nonidet P-40 (NP-40)] and incubated on ice for 5 min. Cells were spun down and supernatants were collected as cytoplasmic fraction. Pelleted nuclei were washed with cytoplasmic lysis buffer and incubated with nucleic lysis buffer (10 mM Tris–HCl pH 7.5, 150 mM NaCl, 3 mM MgCl_2_) for 5 min on ice. Cytoplasmic fraction and pelleted nuclei were treated with Trizol and RNA was extracted from both fractions. Equal volumes of RNA were used for cDNA synthesis.

### LNA-GapmeRs/siRNAs

Cultured HUVECs were transfected at 50–70% confluence with 50 nM LNA-GapmeRs/siRNAs (Qiagen, Hilden, Germany) using Lipofectamine RNAiMax (Life Technologies) according to the manufacturer’s protocol in serum reduced OptiMEM medium (Life Technologies). The medium was changed after 4 h of transfection to ECM. Sequences of the LNA-GapmeRs/siRNAs can be found in the Supplementary Table 2.

### Lentiviral Constructs

Aerrie (NR_038981.1) full length cDNA was cloned into pLenti4/v5 (Life Technologies). Lentivirus stocks were produced in HEK293T cells using pCMVΔR8.91 as packaging plasmid and pMD2.G (Addgene#12259) as vesicular stomatitis virus G glycoprotein envelope expressing plasmid (Zufferey et al., 1997). Empty vectors were used as mock control. Transduction was done for 24 h.

### *In vitro* Sprouting Assay

Human umbilical vein endothelial cells were seeded in ECM containing methylcellulose (20%) into a non-adherent round-bottom 96-well plate to allow one spheroid to be formed per well. Spheroids were collected after 24 h and embedded into a collagen type I gel (BD Biosciences) in a 12-well plate. After polymerization (30 min), ECM was added on top and the plate was incubated for 24 h. Spheroids were fixated with 10% formaldehyde for 15 min and analyzed by bright field microscopy (Olympus IX50, magnification: 10 ×) using the Optimas 6.5 imaging software (Media cybernetics). Cumulative and discontinuous sprout length of each spheroid was measured. Subtraction of the cumulative sprout length from the maximal distance of the migrated cell was defined as discontinuous sprout length. For the rescue experiments with lentiviral Aerrie, HUVECs were stimulated with 50 nM Doxorubicin (Sigma-Aldrich, D1515-10MG) for 2 and 4 h. Equal volumes of DMSO (Sigma-Aldrich, 472301) were used as control.

### Scratch Wound Healing Assay

Cell migration chambers (Ibidi) were placed in a 24-well tissue culture dish. Cells were seeded into each half-chamber and grown overnight. After removal of the inserts, lateral cell migration was visualized by bright field microscopy (EVOS XL AMG, magnification: 4 ×). Pictures were taken at 0 and 6 h after removal of the inserts. Quantitative assay analysis was performed in ImageJ. The area covered by cells was determined for the indicated time points.

### Endothelial Barrier and Wound Healing

Endothelial barrier function was measured by the ECIS system (Applied BioPhysics). 100,000 HUVECs were seeded per well into a gelatin-coated (1%) 10WE plate (Applied BioPhysics). Endothelial barrier integrity was analyzed after 24 h when cells formed a stable monolayer. Barrier resistance (*R*_*b*_) was measured by applying an alternating current of 4000 Hz resulting in a potential which is detected by the ECIS instrument Zθ (Applied BioPhysics), impedance is determined according to Ohm’s law. Cell migration was analyzed by inducing cell wounding through lethal electroporation. Wound repair was observed over a period of 4 h.

### Western Blot Analysis

Human umbilical vein endothelial cells were lysed in Triton X-100 buffer containing benzonase (Santa Cruz Biotechnology, Cas 9025-65-4), protease inhibitors (Thermo Fisher, Halt) and phosphatase inhibitors (Thermo Fisher, Halt) for 1 h on a spinning wheel on 4°C. After centrifugation with 15000 × *g*, protein content was analyzed with Pierce BCA protein assay kit (Thermo Fisher). 10 μg of protein was loaded on Sodium dodecyl sulfate (SDS) gels and blotted on 0.2 μm nitrocellulose membranes (GE healthcare). GAPDH was used as a loading control. Antibodies are listed in the Supplementary Table 3.

### RNA Pulldown

Human umbilical vein endothelial cells were crosslinked with 50 mJ UV light and treated in lysis buffer (50 mM Tris–HCl, 150 mM NaCl, 0.5% NaCl, 0.5% NP-40, 80 U Ribolock protease inhibitor (Thermo Fisher) and volumes were adjusted to 1 ml. Streptavidin-coupled beads C1 (Thermo Fisher) were pre-blocked using ytRNA (Thermo Fisher, AM7119) and glycogen (Thermo Fisher, R0561). For selection of RNP complexes, lysates were pre-cleared with 50 μl pre-blocked beads for 2 h at 4°C and subsequently incubated with 100 pmol 2’O-MeRNA oligonucleotides targeting Aerrie or a scrambled control oligonucleotide for 1 h at 37°C. Sequence of the oligos are listed in the Supplementary Table 2. RNP-oligonucleotide complexes were captured using 25 μl pre-blocked (yeast tRNA, glycogen; both 0.2 mg/ml) streptavidin C1 beads (Thermo Fisher) for 1 h at 37°C. Beads were washed thoroughly with washing buffer (50 mM Tris–HCl pH8, 150 mM NaCl, 0.05% NP-40) and eluted with biotin (250 mM) for 1 h at RT. Eluates were analyzed by RT-qPCR and mass spectrometry.

### Crosslinking RNA Immunoprecipitation

Human umbilical vein endothelial cells were crosslinked with 50 mJ UV light and lysed with total lysis buffer (50 mM, Tris–HCl pH8, 150 mM, NaCl, 0.5% NP-40, and protease inhibitors). The lysates were cleared by centrifugation at 15000 × *g* and incubated with 50 μl protein G magnetic beads (Life Technologies) coated with antibodies listed in the Supplementary Table 3, overnight at 4°C, and then washed with lysis buffer containing 0.05% NP-40. RNA was recovered after protein digestion with Proteinase K (Thermo Fisher, EO0491) by phenol/chloroform/isoamyl extraction and analyzed by RT-qPCR.

### Mass Spectrometry

RNA pulldown eluates were analyzed by liquid chromatography/MS using a QExactive Plus (Thermo Fisher) mass spectrometer equipped with an ultra-high performance liquid chromatography unit (Dionex Ultimate 3000, Thermo Fisher) and a Nanospray Flex Ion-Source (Thermo Fisher). Data analysis was performed in MaxQuant 1.5.3.30, Perseus 1.5.6.0, and plotted in Excel (Microsoft Office).

### Comet Assay

Human umbilical vein endothelial cells were stimulated with 50 nM Doxorubicin (Sigma-Aldrich, D1515-10MG) for 2 and 4 h ECM. As a positive control HUVECs were stimulated with 500 μM H_2_O_2_. Equal volumes of DMSO (Sigma-Aldrich, 472301) were used as control. HUVECs were incorporated in low melting agarose. Cells were lysed with lysis solution (Trevigen, #4250-050-01). DNA was unwinded with alkaline solution (200 mM NaOH, 1 mM EDTA). Electrophoresis was performed at 24V and at least 300 mA for 30 min. Comets were stained with SYBR gold (Thermo Fisher, S11494) for 30 min and visualized with a Zeiss DIMI fluorescent microscope. Comets were analyzed by Cometscore software (RexHoover).

### SCRINSHOT RNA FISH

Subcellular RNA localization was analyzed by SCRINSHOT RNA FISH (Sountoulidis et al., 2020). Briefly, cells were seeded in 1%-gelatin-coated 12-well removable slides (81201, Ibidi) and fixated with 4% PFA after 24 h. Slides were treated with 0.1 M HCl for permeabilization and dehydrated with alcohol to mount hybridization chambers (Grace Bio-labs). After rehydration, the cells were incubated for 30 min with blocking solution containing 0.1 M oligo-dT, Ampligase buffer, 0.05 M KCl, 20% formamide, 0.2 μg/μl BSA, 1 U/μl Ribolock (Thermo Fisher) and 0.2 μg/μl tRNA’s (Ambion). Padlock probes were incubated for 15 min 55°C and 120 min at 45°C, washed with 10% formamide in 2× SSC and ligated with SplintR (NEB) for 16–24 h at 25°C. Rolling circle amplification was performed with Φ29 polymerase (Lucigen) and the primer TAAATAGACGCAGTCAGT^∗^A^∗^A. The amplified products were fixated with 4% PFA and detection oligos were incubated at RT for 60 min. Coverslips were mounted with SlowFade^TM^ Gold Antifade mounting medium.

### Statistical Analysis

Data are expressed as mean ± standard error of the mean (SEM), except the human data from BiKE which is expressed as mean standard deviation. Graphpad Prism 8 was used for statistical analysis. Data were tested with paired or unpaired Student’s *t*-test or Mann-Whitney test when comparing two groups. Analysis of variance (ANOVA) followed by Tukey’s post-test was performed for multiple comparisons. Statistical significance was depicted as follows: ^∗^*P* < 0.05, ^∗∗^*P* < 0.01, ^∗∗∗^*P* < 0.001, ^****^P < 0.0001, ns = not statistically significant.

## Results

### Aerrie Is Expressed in Endothelial Cells and Is Regulated by Aging

**FIGURE 1:**
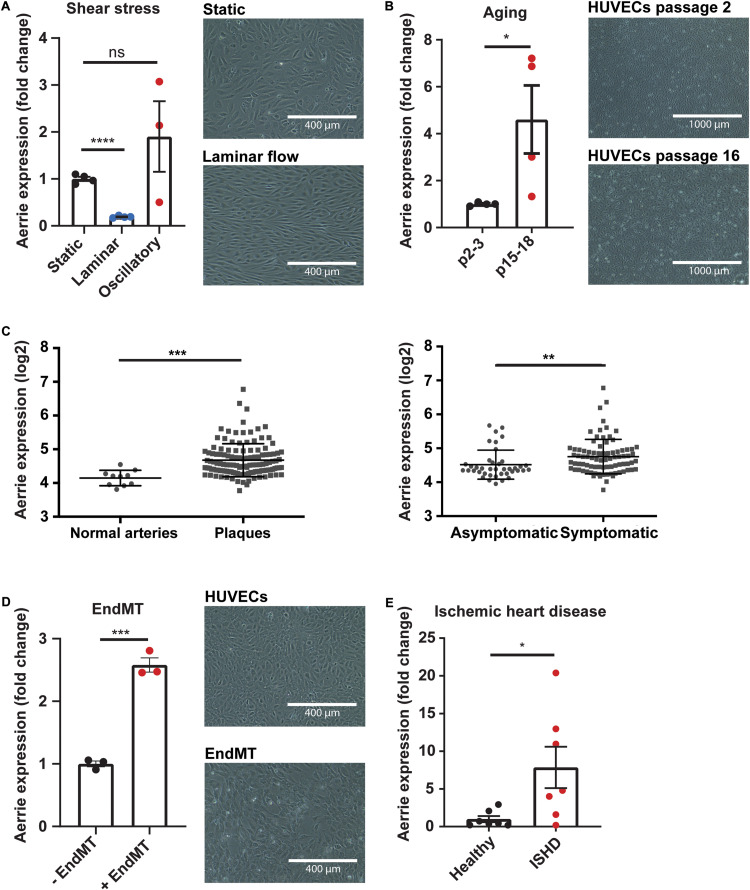
The lncRNA Aerrie is expressed in endothelial cells and is regulated by aging, shear stress, and endothelial-to-mesenchymal transition (endMT). **(A)** Human umbilical vein endothelial cells (HUVECs) were exposed to laminar (72 h, 20 dyn/cm^2^) and oscillatory flow (14 h, 20 dyn/cm^2^). Expression levels of Aerrie were measured by real-time quantitative PCR (RT-qPCR). Expression values are relative to static condition and normalized to GAPDH mRNA (*n* > 3). **(B)** Human umbilical vein endothelial cells (HUVECs) were artificially aged by passing frequently until senescence is reached. Expression levels of Aerrie were measured by RT-qPCR in early and late passages (p) (*n* = 4). **(C)** Human atherosclerotic plaques were collected, and microarray profiled (GSE21545). Expression levels of Aerrie were plotted as normal healthy arteries (*n* = 10) versus atherosclerotic plaques (*n* = 127). From the atherosclerotic plaques, those from asymptomatic (*n* = 40) and symptomatic patients (*n* = 87) were distinguished and expression levels of Aerrie were plotted. **(D)** HUVECs were stimulated with IL-1β and TGF-β2 to induce endMT. Expression levels of Aerrie were measured by RT-qPCR (*n* = 3). **(E)** Human heart tissue of the left ventricle was isolated from healthy donor (*n* = 7) versus ischemic heart disease (ISHD) patients (*n* = 7). Expression levels of Aerrie were measured by RT-qPCR. *p < 0.05; **p < 0.01; ***p < 0.001; ****p < 0.0001; *ns*, not statistically significant.

We identified lncRNA linc01013 in previously published RNA sequencing analysis of HUVECs exposed to laminar shear stress (20 dyn/cm^2^ for 72 h) compared to static conditions (Doddaballapur et al., 2015). Linc01013, which we called Aerrie (Age and EndMT Regulated RNA In Endothelium), is regulated by shear stress (Figure 1A). Aerrie is downregulated under laminar flow while it is upregulated by oscillatory flow. KLF2 is a well-known transcriptional regulator that relays effects of laminar shear stress (Supplementary Figure 1A), accordingly we observed that Aerrie is regulated by KLF2 (Supplementary Figure 1B). Cellular senescence or aging is known to regulate KLF2. In this sense, KLF2 is downregulated by aging in endothelial cells (Boon et al., 2011; Warboys et al., 2014). On the other hand, P21 levels are described to be increased in senescent, aged or disturbed flow exposed endothelial cells (Katsuumi et al., 2018). Therefore, we were interested whether Aerrie is regulated by aging and other age-related diseases, such as atherosclerosis and ischemic heart disease (ISHD). To this end, HUVECs were artificially aged *in vitro* by serial passaging until cells reached senescence. These cells showed increased size, disturbed monolayer formation, and increased expression of Aerrie (Figure 1B). Additionally, we observed that these senescent or “aged” HUVECs have higher P21 levels, indicative of cellular aging (Supplementary Figure 1C). One of the most prominent age-associated vascular diseases, atherosclerosis, is known to occur at sites of flow disturbance in arteries (Wang and Bennett, 2012). We examined the expression levels of Aerrie in normal arteries, asymptomatic and symptomatic atherosclerotic plaques from the BiKE study (Naylor et al., 2003). This analysis showed that Aerrie is increased in atherosclerotic plaques. Additionally, the upregulated expression is also observed in plaques from symptomatic patients compared to asymptomatic atherosclerotic patients (Figure 1C). Another hallmark of vascular aging is endothelial-to-mesenchymal transition (endMT) (Fleenor et al., 2012). To induce endMT, HUVECs are stimulated with IL-1β and TGF-β2 cytokines for 72 h. This treatment resulted in morphological changes, such as increased cell size and elongation, loss of stable monolayer properties (Figure 1D), and increased expression of mesenchymal markers (Supplementary Figure 1D). Importantly, endMT induced the expression of Aerrie (Figure 1D). Ischemic heart disease (ISHD) is common in the older population and therefore of interest for this study (Benjamin et al., 2019). In line with the aging-induced expression of Aerrie, we observed increased expression of Aerrie in ISHD tissue from the left ventricle (Figure 1E). Taken together, these results identify Aerrie as an aging-induced lncRNA.

### Loss of Aerrie Induces Endothelial Dysfunction

**FIGURE 2:**
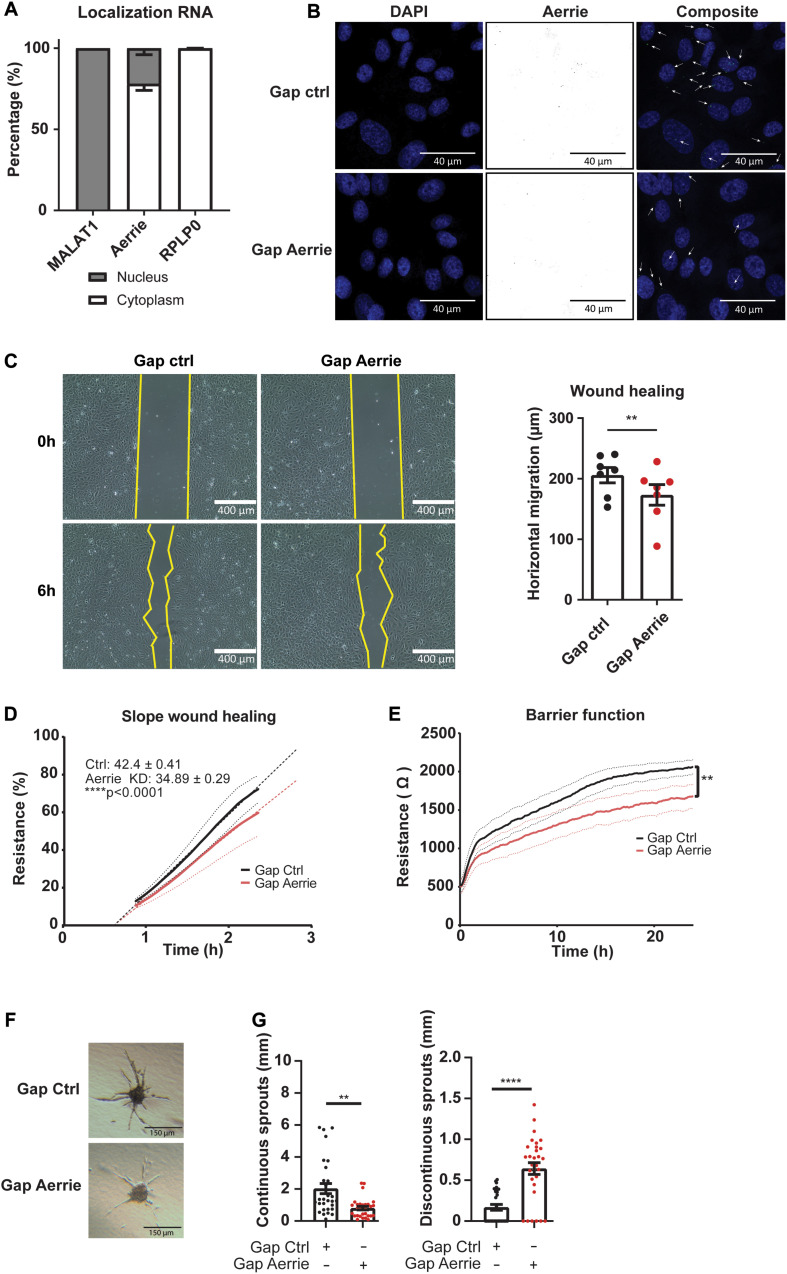
The loss of Aerrie reduces migration, barrier function, and angiogenic sprouting *in vitro*. **(A)** HUVECs were fractionated into nucleus and cytoplasm. RT-qPCR was performed to measure lncRNA Aerrie localization. RPLP0 was used as cytoplasmatic control and lncRNA Malat1 as a nuclear control (*n* = 3). **(B–G)** HUVECs were treated with gapmeR (gap) targeting Aerrie or a respective control. **(B)** Subcellular localization of Aerrie in HUVECs was analyzed by SCRINSHOT RNA FISH. Nuclei were visualized with DAPI. Arrows indicate Aerrie localization. HUVECS were visualized with DAPI on the 405 nm channel and Aerrie on the 488 nm channel. **(C)** Scratch assay was performed with HUVECs for 6 h. The horizontal migration is calculated by the change in surface area (*n* = 7). **(D)** Electric cell-substrate impedance sensing (ECIS) was performed to measure migration of HUVECs. The endothelial monolayer was damaged through lethal electroporation. The recovery slope was determined between 1 to 2 h post wounding (*n* = 3). **(E)** ECIS was performed to measure the barrier function of the endothelial monolayer. The resistance of the endothelial monolayer was determined after 24 h (*n* = 3). **(F)** Brightfield images of silenced Aerrie sprouts and its control. **(G)** Cumulative sprout length was determined for continuous and discontinuous sprouts. (*n* = 3, at least 10 spheroids were measured per experiment). Quantification of the discontinuous sprouts measured by the distance from the tip cell to the stalk cell. **p < 0.01; ****p < 0.0001; *ns*, not statistically significant.

Aging impairs endothelial function such as proliferation and migration and is linked to cellular senescence (Heiss et al., 2005; Moriya and Minamino, 2017). To assess whether reduction of Aerrie contributes to endothelial cell dysfunction, several cellular assays were performed. Firstly, the localization of Aerrie was determined by nuclear and cytoplasmic fractionation. We used RPLP0 as a cytoplasmatic control and the lncRNA Malat1 as a nuclear control. We observed that lncRNA Aerrie is localized in both fractions (Figure 2A). We further confirmed the localization by SCRINSHOT RNA FISH (Figure 2B). To fully deplete both nuclear and cytoplasmic localized Aerrie, we used LNA-GapmeRs, after which Aerrie expression was reduced by 90% (Figure 2B and Supplementary Figure 1E). These oligonucleotides were designed according to our RNA-sequencing data from HUVECs (Supplementary Figure 1F). Depletion of Aerrie impaired HUVEC migration capabilities, as quantified in the scratch assay after 6 h (Figure 2C). This was further validated by performing a wound healing assay by electric cell-substrate impedance sensing (ECIS). Accordingly, loss of Aerrie resulted in slower barrier recovery of the endothelial monolayer after lethal electroporation (Figure 2D). Furthermore, ECIS was performed to assess monolayer integrity by measuring the resistance of the monolayer. Barrier function was significantly decreased upon Aerrie depletion (Figure 2E). Migration and intact cell-cell contacts are important for angiogenic sprouting. Therefore, we assessed the involvement of Aerrie in this process by performing a 3D endothelial cell sprouting assay. Silencing of Aerrie resulted in loss of cumulative sprout length and increased discontinuous sprout length (Figures 2F,G). This suggests that the loss of Aerrie may affect the ability of endothelial stalk cells to follow tip cells during angiogenic sprouting. Taken together, these results show that loss of Aerrie contributes to endothelial dysfunction.

### Depletion of Aerrie Activates DNA Damage Response Mediated by YBX1(2/3)

**FIGURE 3:**
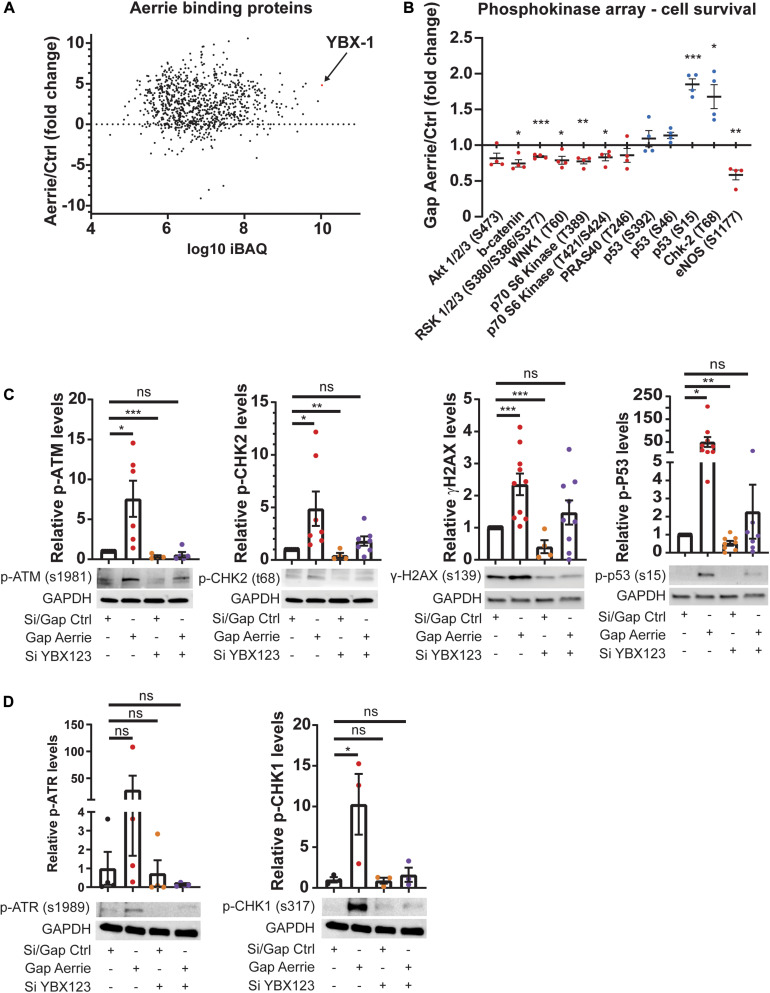
Interaction of Aerrie with YBX1 is important for DNA damage signaling. **(A)** Aerrie-interacting proteins identified by mass spectrometry of RNA-antisense purifications in HUVECs. Identified proteins are plotted against respective IBAQ values. **(B–D)** HUVECs were treated with gapmeR (gap) targeting Aerrie, siRNA (si) targeting YBX1/2/3 or a respective control. **(B)** Phosphokinase levels of cell survival-related proteins (*n* = 4). Red dots are defined as the average level of phosphorylation after silencing knockdown is decreased compared to its respective control. Blue dots are defined as the average level of phosphorylation after silencing knockdown is increased compared to its respective control. **(C)** Phosphorylation levels of activated proteins in the double-strand break repair pathway were analyzed by western blotting. Phosphorylated ATM at serine 1981, CHK-2 at tyrosine 68, H2AX at serine 139, and P53 at serine 15 were analyzed upon depletion of Aerrie and YBX1 (*n* > 4). **(D)** Phosphorylation levels of activated proteins in the single-strand break repair pathway were analyzed by western blotting. Phosphorylated ATR at serine 1989, CHK-1 at serine 317 were analyzed upon depletion of Aerrie and YBX1 (*n* = 3). *p < 0.05; **p < 0.01; ***p < 0.001; *ns*, not statistically significant.

To understand the molecular function of Aerrie in the endothelium, we aimed to identify protein interaction partners of Aerrie. Therefore, we performed an RNA pulldown of endogenous Aerrie followed by protein purification followed by mass spectrometry. Among the many binding proteins, Y-Box protein 1 (YBX1, also known as YB1) had the highest iBAQ value, a measure of absolute protein abundance (Figure 3A; Schwanhausser et al., 2011). The binding of YBX1 to Aerrie was validated by crosslinking RNA immunoprecipitation (CLIP) (Supplementary Figure 2A). YBX1 is a DNA/RNA binding protein that is involved in cell differentiation and embryonal development, stress response, and DNA repair in mammalian cells (Lu et al., 2005; Alidousty et al., 2014; Guarino et al., 2018). However, the exact mechanism of action of YBX1 remains elusive.

**FIGURE 4:**
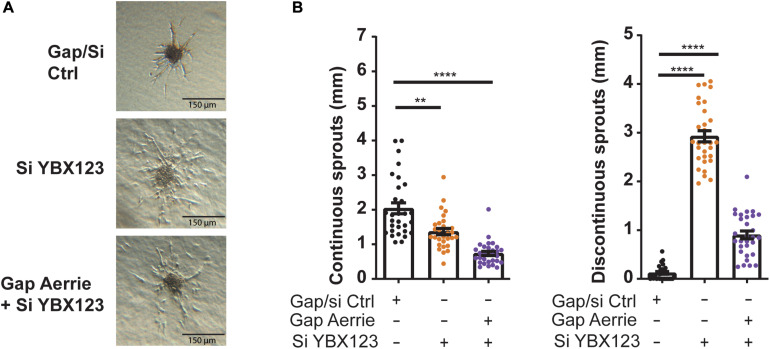
Loss of Aerrie and YBX1/2/3 result in impaired sprouting. **(A,B)** HUVECs were treated with gapmeR (gap) targeting Aerrie, siRNA (si) targeting YBX1/2/3 or a respective control. **(A)** Sprouting assay **(B)** Quantification of the continuous and discontinuous sprouts per condition. (*n* = 3, at least 10 spheroids per experiment). Sprouts measured by the distance from the tip cell to the stalk cell. **p < 0.01; ****p < 0.0001; *ns*, not statistically significant.

To further reveal the molecular function of Aerrie, we decided to profile activation of signaling pathways, using a phosphokinase assay (Figure 3B and Supplementary Figure 2B). A noticeable increase of phosphorylation was observed for P53 and CHK-2, indicating an increase in activation of the DNA damage signaling (Figure 3B). To confirm the activation of these pathways, the phosphorylation levels of the proteins involved in double- and single-strand break repair were determined. Loss of Aerrie showed induced phosphorylation of ATM at serine 1981, CHK-2 at tyrosine 68, H2AX at serine 139, and P53 at serine 15 indicating increased activation of the double-strand break repair (Figure 3C). At the same time increased phosphorylation of proteins involved in single-strand break repair was observed, such as ATR at serine 1989 and CHK1 at serine 317 (Figure 3D). To assess whether YBX1 plays a role in the activation of DNA damage signaling upon loss of Aerrie, we analyzed phosphorylation of DNA repair proteins after silencing YBX1 and its homologs YBX2 and YBX3. The rationale for this is that these homologs might compensate for loss of YBX1 and it is also described that YBX1 can regulate the expression of YBX3 (Lyabin et al., 2020). We indeed observed that a single knockdown of YBX1 is less robust than the knockdown of the entire family (Supplementary Figure 2C). The combination of silenced YBX2 or YBX3 with Aerrie does not result in a robust reduction of phosphorylated ATM compared to the total knockdown of the YBX family (Supplementary Figures 2C, 3C). Therefore, a knockdown of the entire YBX family was performed in our studies. Interestingly, HUVECs without YBX1/2/3 showed no activation of the repair pathway proteins (Figures 3C,D). Moreover, when Aerrie and YBX1/2/3 were co-silenced, the activation of the repair pathway was abolished, indicating a crucial role of YBX1 in DNA repair signaling upon loss of Aerrie. YBX1 is described to be involved in DNA damage, however, its mechanism of action is not known (Kim et al., 2013; Alemasova et al., 2016). As mentioned above, we have shown that loss of Aerrie results in impaired sprouting and that Aerrie is bound to YBX1. To assess whether YBX1/2/3 are involved in endothelial function, we analyzed angiogenic sprouting in endothelial cells with depletion of YBX1/2/3 and Aerrie (Figure 4A). The loss of YBX1/2/3 resulted in similar decrease of sprouting as the inhibition of Aerrie (Figures 2F, 4B). However, discontinuous sprouts were significantly more present (Figure 4B, right). Co-silencing of Aerrie and YBX1/2/3 results in a reduced number of discontinuous sprouts compared to silencing YBX1/2/3 alone. This phenotype is comparable to knockdown of Aerrie alone, suggesting that YBX1 and Aerrie have a common function and that the phenotype can be explained due to inefficient DNA repair during angiogenic sprouting.

### Aerrie Is Involved in DNA Damage and YBX1 Is Required for DNA Damage Signaling

**FIGURE 5:**
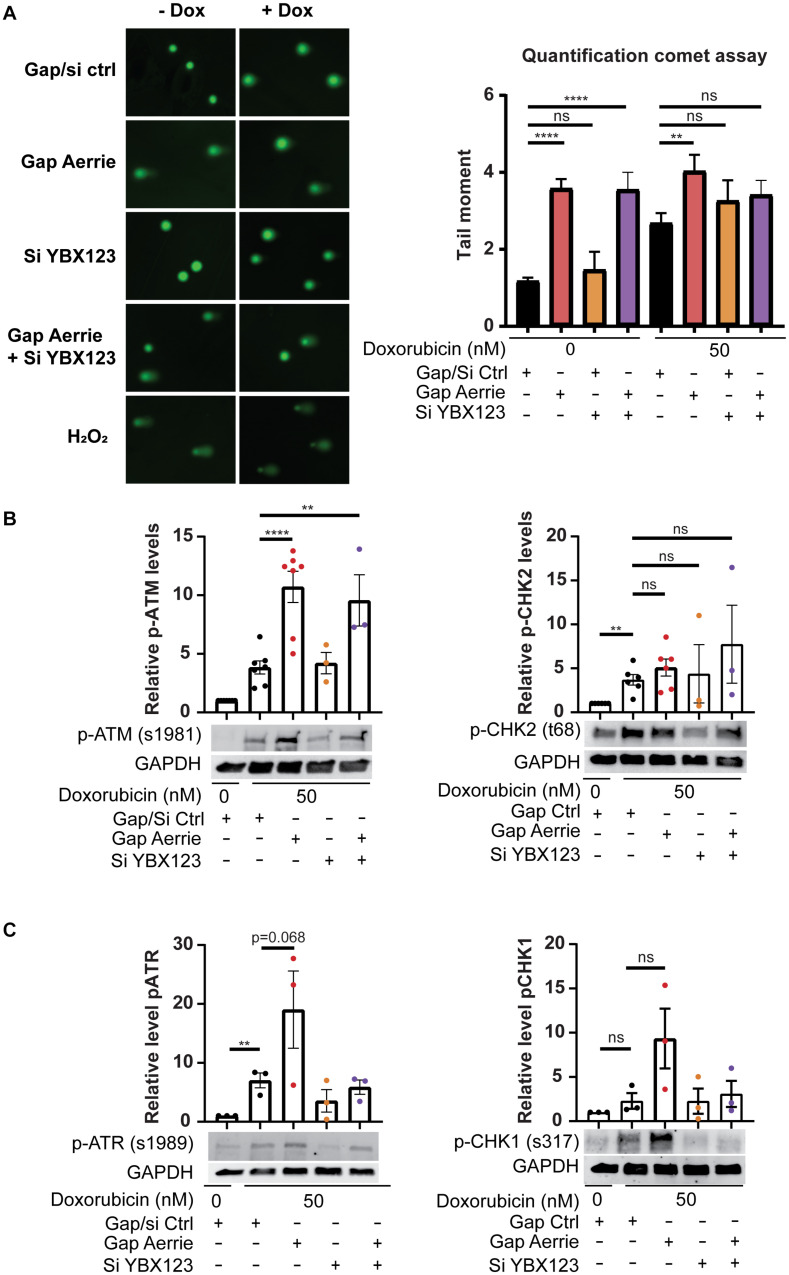
Loss of Aerrie causes DNA damage and activation of signaling, while loss of YBX1/2/3 results in impaired DNA damage signaling. **(A–C)** HUVECs were treated with gapmeR (gap) targeting Aerrie, siRNA (si) targeting YBX1/2/3 or a respective control. **(A)** DNA damage was quantified by comet assay. HUVECs were stimulated with doxorubicin (Dox, 50 nM), embedded in low melting agarose, and stained with SYBR gold for imaging by fluorescence microscopy. Comets were quantified by CometScore (*n* > 3, at least 50 comets per experiment). **(B)** Phosphorylation levels of activated proteins in the double-strand break repair pathway were analyzed by western blotting. Phosphorylated ATM at serine 1981 and phosphorylated CHK-2 at tyrosine 68 were analyzed of Aerrie and YBX1 depleted HUVECs (*n* > 3). **(C)** Phosphorylation levels of activated proteins in the single-strand break repair pathway were analyzed by western blotting. Phosphorylated ATR at serine 1981 and CHK-1 at serine 317 were analyzed of Aerrie and YBX1 depleted HUVECs (*n* = 3). **p < 0.01; ****p < 0.0001; *ns*, not statistically significant.

The loss of Aerrie induces endothelial dysfunction and shows increased DNA damage signaling. Whether only DNA damage signaling is disturbed or whether DNA damage itself is affected is not known. To this end, we performed a comet assay. The comet assay is a well-established assay that can determine the relative amount of DNA damage of cells in all conditions (Olive and Banath, 2006). Doxorubicin is a chemotherapeutical drug that causes the DNA to remain open, damaged and unprotected, leading to apoptosis (Tacar et al., 2013). In line with increased DNA damage signaling upon Aerrie depletion (Figures 3C,D), we observed increased DNA damage upon loss of Aerrie in control and doxorubicin-treated cells (Figure 5A). However, whereas silencing YBX1 rescues DNA damage signaling induced by loss of Aerrie (Figures 3C,D), DNA damage itself is not affected by silencing YBX1. This indicates that loss of Aerrie induces DNA damage (and subsequent signaling), and that YBX1 is required only for subsequent signaling and is not involved in DNA damage induction after loss of Aerrie. Furthermore, DNA damage signaling in HUVECs treated with Doxorubicin is increased in all conditions (Figures 5B,C), as assessed by western blotting. Despite of the silencing of the YBX family, DNA damage signaling is still activated by doxorubicin. This effect can be explained by other DNA damage signaling activation that bypasses Aerrie and YBX1 (Paull, 2015). Taken together, the data suggests that knockdown of Aerrie induces DNA damage, and that DNA damage repair in turn requires Aerrie and YBX1 for efficient signaling to repair the DNA.

### Lentiviral Overexpression of Aerrie Improves DNA Repair and Angiogenic Sprouting

**FIGURE 6:**
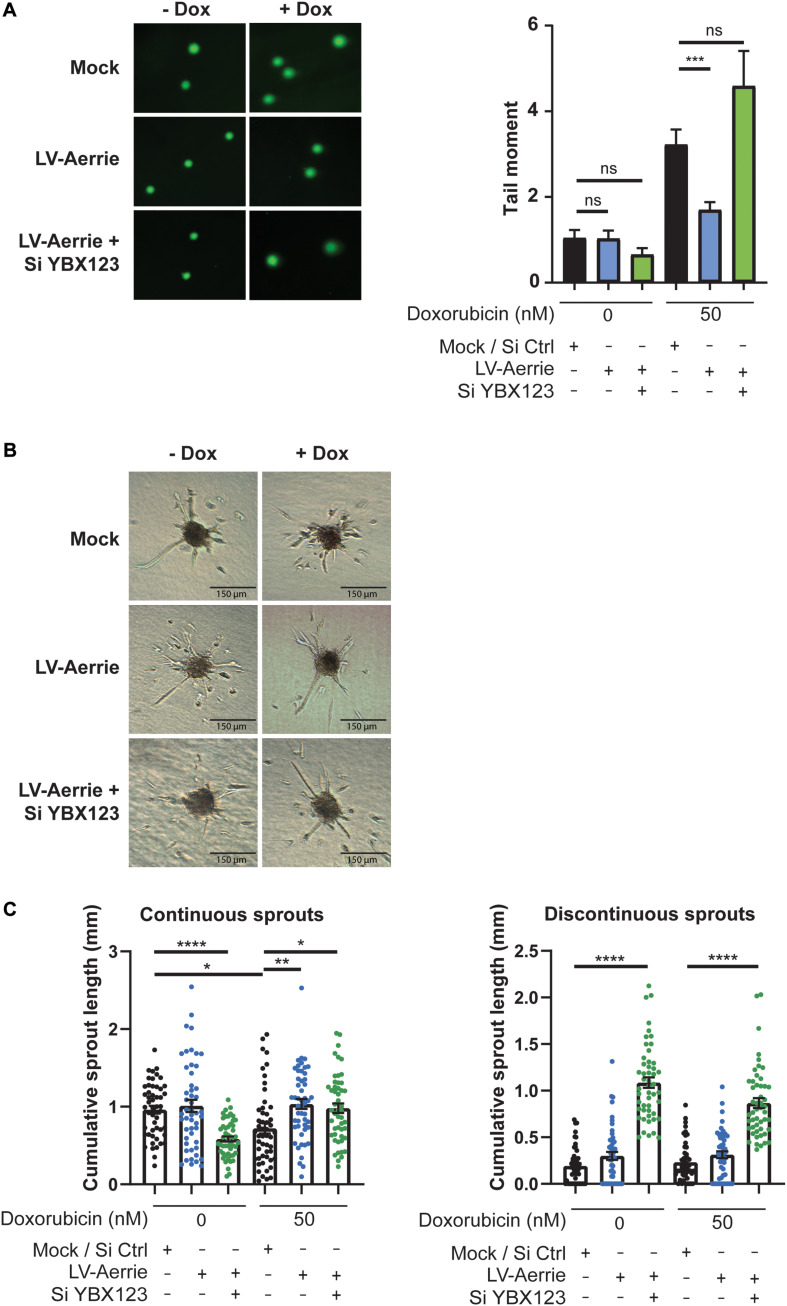
Overexpression of Aerrie improves DNA repair and angiogenic sprouting and is impaired by loss of YBX1/2/3. **(A–C)** HUVECs were treated with lentivirus (LV) for Aerrie overexpression and siRNA (si) targeting YBX1/2/3 or a respective control. **(A)** HUVECs with depleted Aerrie or YBX1 are stimulated with Doxorubicin (50 nM) and embedded in low melting agarose stained with SYBR gold and imaged by fluorescence microscopy. Comets were quantified by CometScore. (*n* > 3, at least 50 comets per experiment) **(B)** Sprouting assay performed with or without stimulation of Doxorubicin (50 nM). **(C)** Quantification of the continuous and discontinuous sprouts per condition. Measured by the distance from the tip cell to the stalk cell. (*n* = 3, ≥10 spheroids per experiment). *p < 0.05; **p < 0.01; ***p < 0.001; ****p < 0.0001; *ns*, not statistically significant.

Next, we determined whether Aerrie gain-of-function inhibits DNA damage and induces angiogenic sprouting. Therefore, we overexpressed Aerrie by lentiviral transduction. Without an exogenous DNA damage stimulus, Aerrie overexpression does not affect DNA damage, as measured by comet assay (Figure 6A). Inducing DNA damage using doxorubicin increases the tail moment by two-fold and, interestingly, is reduced upon overexpression of Aerrie (Figure 6A). Indeed, overexpression of Aerrie reduces DNA damage and suggests that DNA repair is enhanced. This effect is diminished when YBX1/2/3 is knocked down, implying that YBX1 and Aerrie together are necessary to have efficient DNA repair.

To identify whether impaired endothelial sprouting after silencing Aerrie is caused by defect DNA repair (Figure 2F), we stimulated endothelial spheroids overexpressing Aerrie with doxorubicin. Indeed, overexpression of Aerrie show improved angiogenic sprouting under doxorubicin stimulation compared to mock (Figures 6B,C). Unexpectedly, we also observed improved continuous sprouts when YBX1/2/3 is knocked down in combination with Aerrie overexpression. However, we did not observe a reduction of discontinuous sprouts in the same condition, suggesting that Aerrie overexpression is unable to rescue induction of discontinuous sprouts in the absence of YBX1/2/3. The increase of discontinuous sprouts by loss of YBX1/2/3 is the same effect we have observed in the previous experiment (Figures 4A,B). A possible explanation for this phenotype is a defect in proliferation on the long term (Kotake et al., 2017).

**FIGURE 7:**
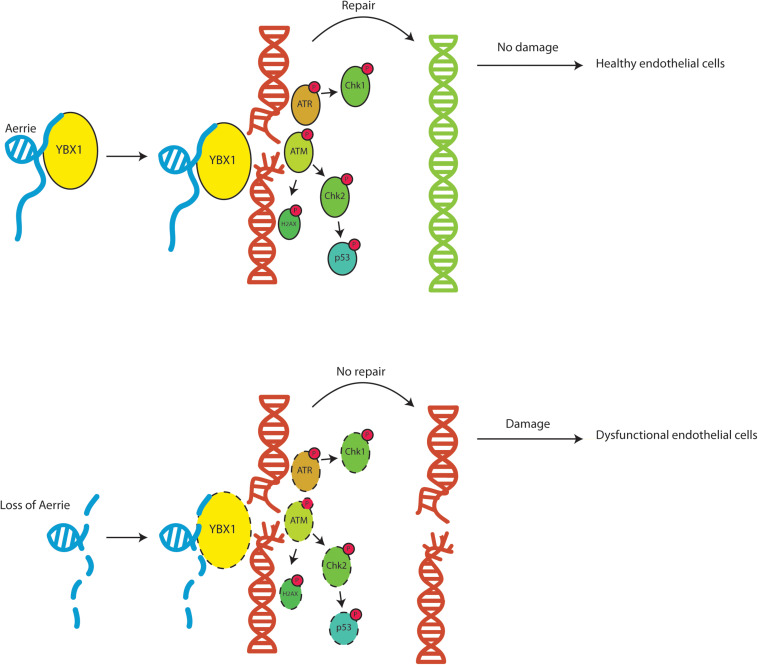
Schematic overview of the function of lncRNA Aerrie and YBX1. Aerrie as an important factor in genomic stability and as a binding partner to YBX1 in responding to genomic stress in endothelial cells.

In summary, we show that the novel lncRNA Aerrie is required for normal endothelial function. Aerrie is upregulated by aging which may have implications for CVD. We show that depletion of Aerrie causes detrimental effects that results in impaired sprouting, migration, and barrier function. HUVECs overexpressing Aerrie are less prone to DNA damage suggesting more efficient DNA repair. Additionally, Aerrie interacts with YBX1 and upon depletion of YBX1 and Aerrie, DNA damage signaling is impaired and increased DNA damage is observed. The interaction of Aerrie and YBX1 is needed for efficient DNA repair to maintain proper angiogenic sprouting. In conclusion, lncRNA Aerrie and YBX1 are important factors in DNA damage repair in endothelial cells that may be needed to maintain cardiovascular homeostasis (Figure 7).

## Discussion

Our results demonstrate that the novel lncRNA Aerrie is an essential regulator of endothelial DNA repair and that Aerrie expression is increased during aging. Loss of Aerrie is accompanied by impaired angiogenic sprouting, migration, and endothelial barrier function. Overexpression of Aerrie improved DNA repair efficiency when the genome is damaged by doxorubicin. YBX1 is an important binding partner of Aerrie and it is essential to activate signaling of key components of the DNA repair pathway (Figure 7).

Aerrie expression is increased with aging, disturbed flow, and endMT *in vitro* (Figures 1A,B,D). Aerrie is expressed in arteries and the heart and is increased in atherosclerotic plaques and in ischemic hearts (Figures 1C,E). However, the regulation of Aerrie by aging remains elusive. KLF2 is known to be regulated by shear stress and acts as an atheroprotective factor (Fledderus et al., 2007). Mechanistically, we observe that KLF2 regulates Aerrie expression (Supplementary Figure 1B). Overexpression of KLF2 decreases expression of Aerrie. Accordingly, we observe repressed expression of Aerrie *in vitro* under laminar flow (Figure 1A) when KLF2 expression is increased (Supplementary Figure 1A), while Aerrie expression is increased under disturbed flow when KLF2 is decreased (Figure 1A and Supplementary Figure 1A). Future studies will address the potential role of KLF2 in controlling Aerrie expression during aging. Another mechanism that regulates Aerrie is endMT. We identify JMJD2B, a recently reported factor involved in endMT (Glaser et al., 2020), as a potential regulator of Aerrie expression (Supplementary Figure 2D), but whether JMJD2B is involved in endothelial aging remains unknown.

Aerrie or LINC01013 has already been studied in Anaplastic Large-Cell Lymphoma (ALCL). Chung et al. (2017) describes Aerrie as an invasion activator of epithelial-to-mesenchymal transition (EMT). EMT is a physiological process for embryonic development, but is also found to play a role in fibrosis and cancer (Stone et al., 2016). EndMT is described as an analogous process in endothelial cells sharing similar cellular and molecular events (Medici and Kalluri, 2012). In both processes, the cells lose their adherens junctions and increase mesenchymal markers to transition into a mesenchymal phenotype. Chung et al. has shown that Aerrie regulates Snail to promote ALCL invasion. However, we observed that Snail is not regulated by Aerrie in HUVECs (Supplementary Figure 2E). Suggesting that Aerrie have different functions in other cell types.

Aging is a complex process that has multiple effects on the endothelium. One of the hallmarks of aging is increased DNA damage (Donato et al., 2015). Our data show that loss of Aerrie results in DNA damage and markers such as ATM, CHK2, γH2AX, P53, ATR, CHK1 are activated. Interestingly, overexpression of Aerrie enhances the DNA repair capabilities and results in less DNA damage when stimulated with doxorubicin (Figure 6A). The model that emerges from these data is that an increase in Aerrie expression with aging likely contributes to protection from endothelial dysfunction, but this rescues mechanism is not sufficient to prevent aging-induced endothelial dysfunction.

We showed that Aerrie controls DNA repair *via* YBX1 interaction. Loss of YBX1 reduces efficient DNA repair even in the presence of Aerrie (Figure 6A). YBX1 has a broad function in various cell types and is recognized as an oncogenic factor in several tumors (Lim et al., 2019; Kim et al., 2020). YBX1 participates in post-transcriptional regulation, stress response signaling, inflammation, and DNA damage (Guarino et al., 2018; Wang et al., 2019; Hermert et al., 2020). YBX1 can bind to the DNA close to the damage site with its cold shock domain (Zhang et al., 2020). Together with Aerrie, this may be necessary to recruit DNA damage repair proteins to ensure proper DNA damage repair, such as ATM and ATR. It is unclear whether and how the Aerrie-YBX1 complex recruits the DNA repair proteins. However, reduction of YBX1 was sufficient to abolish the activation of ATM, CHK2, P53, γH2AX, ATR, and CHK1 in order to repair DNA damage (Figures 3B,C, 5B,C). A possible mechanism would be that upon loss of Aerrie, YBX1 cannot be translocated to the nucleus (Koike et al., 1997; Das et al., 2007). Without the translocation, DNA repair proteins cannot be recruited or activated by Aerrie and YBX1. Slightly higher expression of YBX1 is observed upon knockdown of Aerrie, although the localization is not known (Supplementary Figure 2F). For example, it is described that the lncRNA TP53TG1 blocks the translocation of YBX1 to the nucleus to repair the DNA (Diaz-Lagares et al., 2016). The study provides evidence that epigenetic silencing of TP53TG1 results in increased DNA damage resistance by YBX1 and the activation of the PI3K/AKT pathway.

Mechanistically, YBX1 is known to be phosphorylated by CHK1 at threonine 80 (Blasius et al., 2011). We have detected an increase in phosphorylated CHK1 and other DNA damage markers with loss of Aerrie. A possible explanation is that CHK1 regulates YBX1 and thereby regulates DNA repair. Dissociation of YBX1 from the DNA repair mechanism can be achieved by ubiquitination. It has been described that YBX1 can be ubiquitinated by RBBP6 and its RING finger domain (Chibi et al., 2008). Thus, dissociation of YBX1 from the DNA repair machinery may be achieved by ubiquitination. LncRNA Aerrie and YBX1 may be involved in a more complex and versatile system than we have explored. A possible mechanism could be that YBX1 and Aerrie are being recruited by DNA damage sensing proteins such as ATM to enhance the DNA damage signaling. In these events, ATM is capable to modulate the function of CHK1 and CHK2 to slow down the cell cycle (Awasthi et al., 2015). CHK1 subsequently activates YBX1 to complement the DNA damage signaling to repair the DNA. Upon knockdown of YBX1, the DNA repair signaling would not be activated when DNA damage is present, which fits to our data.

Surprisingly, we discovered that overexpression of Aerrie and the loss of YBX1/2/3 results in rescue of continuous sprouts under stimulation of doxorubicin (Figures 6B,C). No rescue in the sprouting assay was expected due to the increase of DNA damage in the comet assay (Figure 6A). It is possible that the reduction of DNA damage signaling by loss of YBX1 and the inhibition of DNA damage by overexpressed Aerrie the cells can survive apoptosis for a short period of time. In this sense, the cells can undergo angiogenic sprouting and proliferation until the threshold of accumulated DNA damage is reached. Also, the amount of error prone repair can reach a limit until the cells undergo apoptosis (Khanna, 2015). With the same thought, we could argue that the rescue of continuous sprouts that we observe (Figures 6B,C) is due to accumulated amount of error-repaired DNA that results in cancerous cell growth.

In conclusion, this study provides evidence that lncRNA Aerrie is required for proper DNA damage signaling, via interaction YBX1, and for efficient DNA repair in endothelial cells.

## Data Availability Statement

Publicly available datasets were analyzed in this study. This data can be found here: https://www.ncbi.nlm.nih.gov/geo/query/acc.cgi?acc=GSE21545.

## Ethics Statement

The studies involving human participants were reviewed and approved by the Stockholm Regional Ethics Committee. The patients/participants provided their written informed consent to participate in this study.

## Author Contributions

TP designed and performed the experiments, analyzed the data, and drafted the manuscript. DB provided technical, conceptual advice, and performed the experiments. LS provided technical, conceptual advice, and provided samples. AB, EL, and YT performed the experiments. LM and UH provided data. IW performed the mass spectrometry measurements and analysis. SD provided data and gave conceptual advice. RB supervised the project, designed experiments, analyzed data, handled funding, and drafted the manuscript. All authors contributed to the article and approved the submitted version.

## Conflict of Interest

The authors declare that the research was conducted in the absence of any commercial or financial relationships that could be construed as a potential conflict of interest.

## Supplementary Material

Supplementary Figure 1 **(A)** KLF2 mRNA expression in HUVECs stimulated under static, laminar (20 dyn/cm^2^ for 72 h), or oscillatory flow (20 dyn/cm^2^ for 14 h). Expression levels of Aerrie were measured by real-time quantitative PCR (RT-qPCR). Expression values are relative to static condition and normalized to GAPDH mRNA (*n* > 3). **(B)** RNA expression levels of Aerrie and KLF2 measured in lentiviral KLF2 induced HUVECs by RT-qPCR. Expression values are normalized to RPLP0 RNA. **(C)** P21 RNA expression levels in early vs. late passaged HUVECs by RT-qPCR. Expression values are normalized to RPLP0 RNA. **(D)** EndMT markers SM22, snail, CNN1 expression levels of HUVECs and endMT induced HUVECs measured by RT-qPCR. Expression values are normalized to RPLP0 RNA. **(E)** Expression level of Aerrie after knockdown with LNA-GapmeR measured by RT-qPCR. Expression values are normalized to RPLP0 RNA. **(F)** RNA-sequencing of HUVECs at Aerrie region. The vertical lines indicate reads and the arches above and below indicate splice junctions. 4 exons of 2 known validated transcripts of Aerrie annotated in NCBI show reads, namely NR_038981.1, and NR146223.1.

Supplementary Figure 2 **(A)** RT-qPCR of Aerrie from the elution of RNA crosslinking immunoprecipitation (CLIP) experiments of YBX1. **(B)** Quantified phosphorylation levels of proteins from the phosphokinase assay. **(C)** Phosphorylation levels of activated ATM after separate YBX1, YBX2, and YBX3 knockdowns analyzed by western blotting. **(D)** EndMT was induced by IL-1β and TGF-β2. Aerrie expression level were measured by RT-qPCR after JMJD2B knockdown in endMT stimulated HUVECs. Expression values are normalized to RPLP0 RNA. **(E)** Snail RNA expression levels in HUVECs by RT-qPCR. HUVECs were treated gapmeR targeting Aerrie (gap) or a respective control. Expression values are normalized to RPLP0 RNA. **(F)** YBX1 levels measured upon knockdown of Aerrie by western blotting (*n* > 3).

Supplementary Table 1 ISHD patient IDs with their corresponding information. Left ventricular tissues from ISHD samples were acquired from the University of Sydney (Sydney, NSW, Australia), with the ethical approval of the Human Research Ethics Committee (number 2012/2814). Explanted left ventricular heart tissue of healthy donors was used as control samples; the donors died from a non-cardiac cause, typically motor vehicle accidents. The healthy donor samples were also acquired from the University of Sydney.

Supplementary Table 2 Gene silencing, qPCR primer and RNA pulldown oligo sequences. (ST3) Antibody list used for Western blots and RNA IPs.
