# Supplementary material for: Long Non-coding RNA Aerrie Controls DNA Damage Repair via YBX1 to Maintain Endothelial Cell Function
Source: Front Cell Dev Biol. 2021 Jan 11;8:619079. doi: 10.3389/fcell.2020.619079 (PMC7829583; doi:10.3389/fcell.2020.619079)

Supplement 1

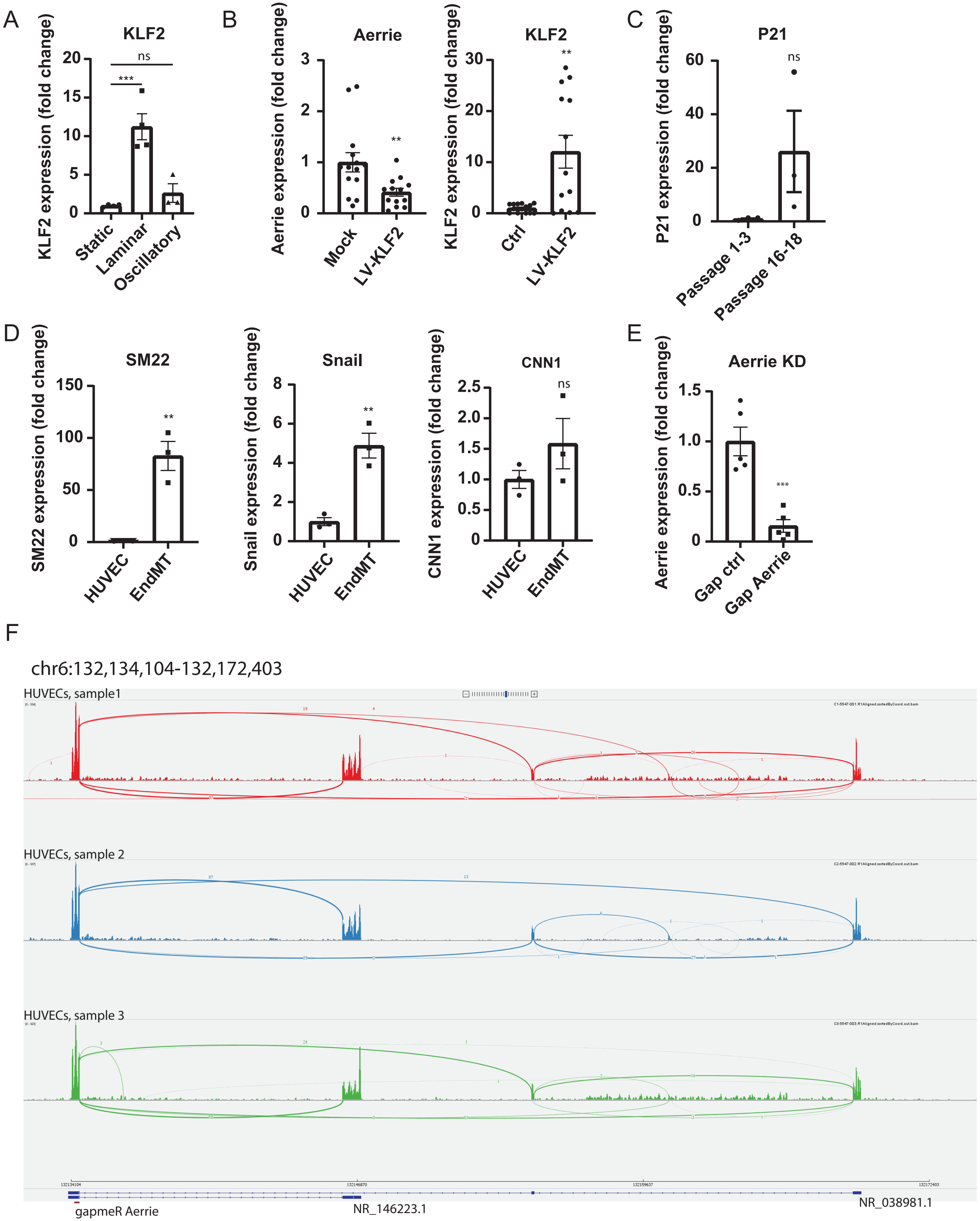

Supplement 2

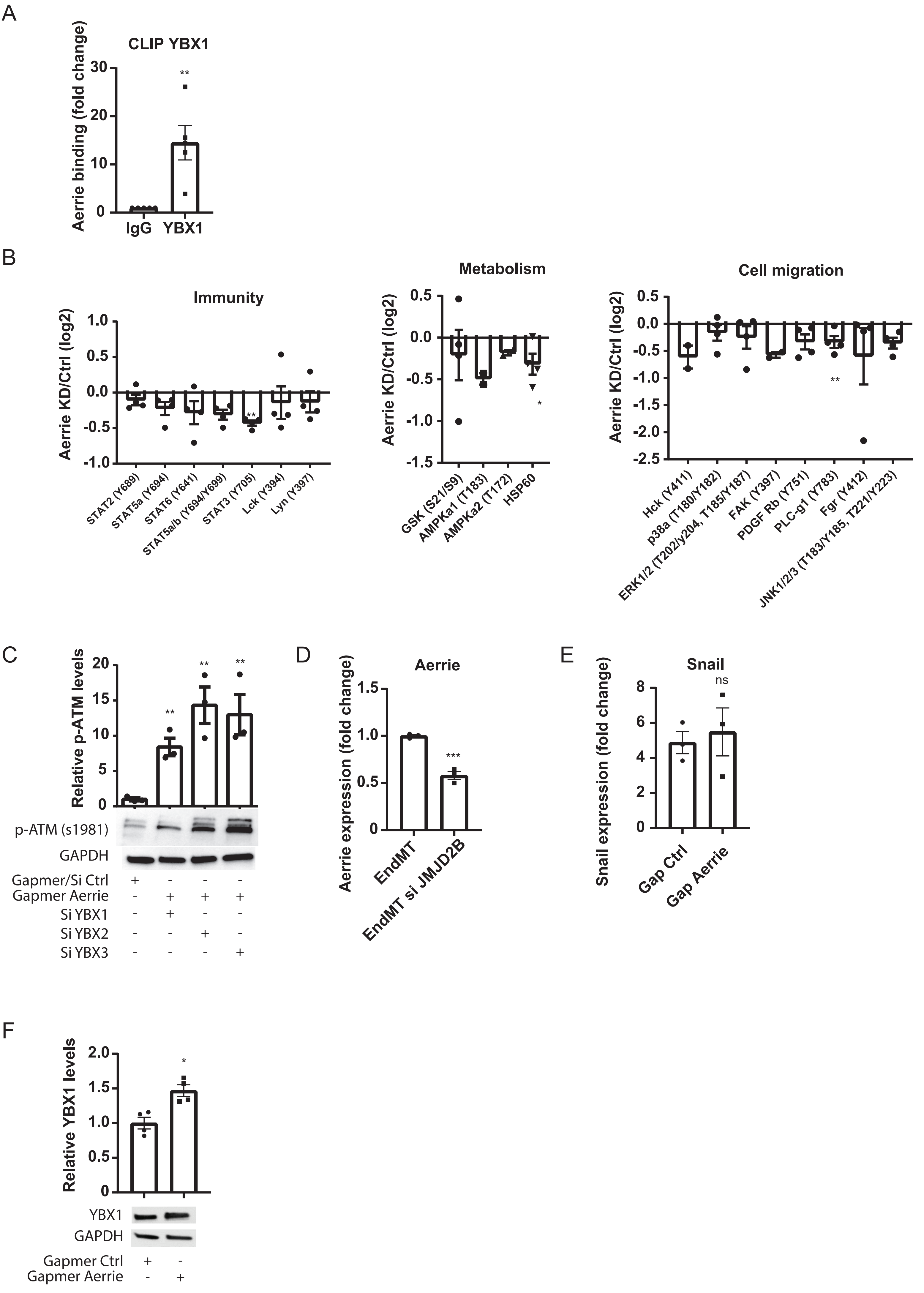

Supplement: Supplementary Figure 1 — (A) KLF2 mRNA expression in HUVECs stimulated under static, laminar (20 dyn/cm2 for 72 h), or oscillatory flow (20 dyn/cm2 for 14 h). Expression levels of Aerrie were measured by real-time quantitative PCR (RT-qPCR). Expression values are relative to static condition and normalized to GAPDH mRNA (n > 3). (B) RNA expression levels of Aerrie and KLF2 measured in lentiviral KLF2 induced HUVECs by RT-qPCR. Expression values are normalized to RPLP0 RNA. (C) P21 RNA expression levels in early vs. late passaged HUVECs by RT-qPCR. Expression values are normalized to RPLP0 RNA. (D) EndMT markers SM22, snail, CNN1 expression levels of HUVECs and endMT induced HUVECs measured by RT-qPCR. Expression values are normalized to RPLP0 RNA. (E) Expression level of Aerrie after knockdown with LNA-GapmeR measured by RT-qPCR. Expression values are normalized to RPLP0 RNA. (F) RNA-sequencing of HUVECs at Aerrie region. The vertical lines indicate reads and the arches above and below indicate splice junctions. 4 exons of 2 known validated transcripts of Aerrie annotated in NCBI show reads, namely NR_038981.1, and NR146223.1. [file Data_Sheet_3.pdf]
