# Supplementary material for: Long Non-coding RNA Aerrie Controls DNA Damage Repair via YBX1 to Maintain Endothelial Cell Function
Source: Front Cell Dev Biol. 2021 Jan 11;8:619079. doi: 10.3389/fcell.2020.619079 (PMC7829583; doi:10.3389/fcell.2020.619079)
Supplement: Supplementary Table 1 — ISHD patient IDs with their corresponding information. Left ventricular tissues from ISHD samples were acquired from the University of Sydney (Sydney, NSW, Australia), with the ethical approval of the Human Research Ethics Committee (number 2012/2814). Explanted left ventricular heart tissue of healthy donors was used as control samples; the donors died from a non-cardiac cause, typically motor vehicle accidents. The healthy donor samples were also acquired from the University of Sydney. [file Data_Sheet_1.PDF]

Supplement

ST1

Patient ID	Sex	Age	Category
4091	M	53	HF 5y;Renal failure,Dx 5years,x3 CABG,psorisis;AF;elevated cholesterol;plmonary hyperplaysia,LAD 100%;Graft 70%;LCX 50%;RCA graft 100%;APEX ischemic & endocardium is more ischemic;
4093	M	55	LV 6y 3xCABG; hypothyroid;diabetes; AF;fam Hx. MYHA Class III,3CABG,AF,LV systolic impairment. FHx
5068	M	49	*Global Ischemia, Multiple Stents*
4070	M	50	LAD total
4074	M	61	Triple CABG;Circumfl 70%, smoked 12 years,social drinker,angioplasty, multiple adhesions.Drugs: Warfrin, amiodarome,digoxin,fusemide,temazapan, puride.
4076	M	62	10y AMI 94;Recurrent AF;:Alcohol 10-20g/day;smoker.Anterior;Atrial reduction;Ovarectamy 03;
4108	M	62	CABG; ICD implant;IHD,CCF,ICD implant
4062	M	55	Xs inotropes;bad coron aa; grade IV SAH, CVD? Hi WBC drugs coron aa data
3141	M	52	ICH
3145	M	39	MVA with bruising
4095	F	48	Grade V SAH
4104	F	59	LA/RA/RV; Cor aa
