# Supplementary material for: Long Non-coding RNA Aerrie Controls DNA Damage Repair via YBX1 to Maintain Endothelial Cell Function
Source: Front Cell Dev Biol. 2021 Jan 11;8:619079. doi: 10.3389/fcell.2020.619079 (PMC7829583; doi:10.3389/fcell.2020.619079)
Supplement: Supplementary Table 2 — Gene silencing, qPCR primer and RNA pulldown oligo sequences. (ST3) Antibody list used for Western blots and RNA IPs. [file Data_Sheet_2.PDF]

Supplement

ST2

Gene silencing oligos	Sequence (5'-3')
Gapmer ctrl	AACACGTCTATACGC
Gapmer Aerrie	GTCTTAACTGGAAGAA
Si Ctrl	MISSION siRNA Universal Negative Control #1 (SIC001)
Si YBX1	CCUAUGGGCGUCGACCACA[dT][dT]
	UGUGGUCGACGCCCAUAGG[dT][dT]
Si YBX2	CCGUGAAGGGCAGCCGUUA[dT][dT]
	UAACGGCUGCCCUUCACGG[dT][dT]
Si YBX3	CGCCUUACCACGUGGGACA[dT][dT]
	UGUCCCACGUGGUAAGGCG[dT][dT]
**RT-qPCR primer oligos**	
Aerrie	AAGCCAGCATTTTTAATAAATAGGAA
	AAACTGCCTCCTCAGACTGC
RPLP0	TCGACAATGGCAGCATCTAC
	ATCCGTCTCCACAGACAAGG
KLF2	CAAGACCTACACCAAGAGTTCG
	CATGTGCCGTTTCATGTGC
Malat-1	GTGATGCGAGTTGTTCTCCG
	CTGGCTGCCTCAATGCCTAC
YBX1	GGAGTTTGATGTTGTTGAAGGA
	AACTGGAACACCACCAGGAC
YBX2	CCCTACCCAGTACCCTGCT
	CCTTCCTTCAACCCTTGATAA
YBX3	CGGTTCATCGAAATCCAACT
	TAATTGTAGGGACGCCGGTA
**RNA pulldown oligos**	
Scrambled Ctrl	GGACGATTCGATCGATAATCT
Aerrie	AATAATACTTGGGCACTGCTG

ST3

Antibody	Species	Company	Catalog #	Application	Dilution
GAPDH	Rabbit	Cell signaling	14C10	WB	10000
p-p53 (s15)	Rabbit	Cell signaling	9284S	WB	1000
p-ATM (s1981)	Rabbit	Abcam	ab81292	WB	500
γH2AX (s139)	Mouse	Merck	05-636	WB	1000
p-Chk2 (t68)	Rabbit	Cell signaling	2661S	WB	1000
p-ATR (T1989)	Rabbit	Cell signaling	58014S	WB	500
p-Chk1 (s317)	Rabbit	Cell signaling	2344S	WB	1000
anti-mouse HRP	Goat	Dako	p0447	WB	5000
anti-rabbit HRP	Goat	Dako	p0448	WB	5000
YBX1	Rabbit	Cell signaling	D2A11	RIP	3 ug
IgG	Rabbit	Cell signaling	31887	RIP	3 ug
